# Recurring cluster and operon assembly for Phenylacetate degradation genes

**DOI:** 10.1186/1471-2148-9-36

**Published:** 2009-02-10

**Authors:** Fergal J Martin, James O McInerney

**Affiliations:** 1 Department of Biology, National University of Ireland Maynooth, Maynooth, Co. Kildare, Ireland

## Abstract

**Background:**

A large number of theories have been advanced to explain why genes involved in the same biochemical processes are often co-located in genomes. Most of these theories have been dismissed because empirical data do not match the expectations of the models. In this work we test the hypothesis that cluster formation is most likely due to a selective pressure to gradually co-localise protein products and that operon formation is not an inevitable conclusion of the process.

**Results:**

We have selected an exemplar well-characterised biochemical pathway, the phenylacetate degradation pathway, and we show that its complex history is only compatible with a model where a selective advantage accrues from moving genes closer together. This selective pressure is likely to be reasonably weak and only twice in our dataset of 102 genomes do we see independent formation of a complete cluster containing all the catabolic genes in the pathway. Additionally, *de novo *clustering of genes clearly occurs repeatedly, even though recombination should result in the random dispersal of such genes in their respective genomes. Interspecies gene transfer has frequently replaced *in situ *copies of genes resulting in clusters that have similar content but very different evolutionary histories.

**Conclusion:**

Our model for cluster formation in prokaryotes, therefore, consists of a two-stage selection process. The first stage is selection to move genes closer together, either because of macromolecular crowding, chromatin relaxation or transcriptional regulation pressure. This proximity opportunity sets up a separate selection for co-transcription.

## Background

**Figure 1:**
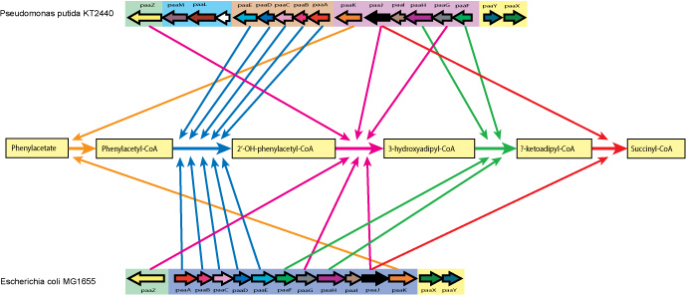
**Schematic for the degradation of phenylacetate, including genes involved and the cluster and operon structures in *E. coli K12 *and *P. putida KT2440***.

The aerobic degradation of phenylacetic acid in *E. coli K12 *occurs *via *a series of five reactions, involving eleven catabolic *paa *genes [1], two of which are distant paralogs, with the rest showing no sequence homology (figure 1). The first step of the pathway is catalysed by the product of the *paaK *gene, a CoA ligase that catalyses the conversion of phenylacetate into phenylacetyl-CoA. The second step involves a ring-oxygenase complex formed from the gene products of *paaABCDE*. This heteromer converts phenylacetyl-CoA into 2'-OH-phenylacetyl-CoA. The third step, where 2'-OH-phenylacetyl-CoA is converted to 3-hydroxyadipyl-CoA, is jointly catalysed by *paaJ*, *paaG *and *paaZ*. The fourth step sees the conversion of 3-hydroxyadipyl-CoA by *paaF *and *paaH *to β-ketoadipyl-CoA. The final step is catalysed by *paaJ*, which converts β-ketoadipyl-CoA to succinyl-CoA, thereby connecting phenylacetate degradation with the TCA cycle [1]. In addition to these 11 catabolic genes, *E. coli K12 *has 3 other *paa *genes, two of which regulate the pathway (*paaX *and *paaY*), the other has an unknown function (*paaI*). Other *E. coli *strains such as *E. coli O157 *and *E. coli O73 *do not share homologs to all 11 catabolic genes, with no homologs found for *paaA*, *paaB*, *paaC*, *paaD, paaE, paaG *and *paaK *in either of these two genomes. However, previous studies have identified other bacteria as having homologs to *paa *genes, such as *Pseudomonas putida U *[2]. In addition to these 14 genes found in *E. coli K12*, a further three genes associated with the pathway were examined in this study. These were *paaL *and *paaM*, coding for a phenylacetic acid transporter protein and a phenylacetic acid specific porin respectivelty, and *tetR*, a transcription factor.

The genes involved in phenylacetate degradation in *E. coli K12 *and *P. putida U *are located in clusters [2,3]. In this study we define a gene cluster as a set of functionally related genes located in close physical proximity in a genome. The term operon refers to a set of genes under common regulatory control, that are transcribed into a single mRNA and are all co-directional in orientation on the chromosome. An operon, therefore, is a more structured instance of a cluster. All operons by definition are also clusters, but not all clusters are operons. A gene cluster can consist entirely of independently transcribed genes or multiple operon structures or combinations of both. Clusters and operons are observed both in prokaryotes and eukaryotes, however, the system of operon processing in eukaryotes involves mRNA splicing, and is different to the system in prokaryotes [4,5].

Clustering of genes involved in the same metabolic pathway is a widespread phenomenon [6-10], and the polycistronic operon is a paradigm of prokaryotic genomic biology [11]. However, the process of operon formation remains poorly understood and the precise link between clustering and operon formation has never been fully explained, though several models exist.

The simplest model is the Natal Model where clusters form via tandem gene duplications [12]. However, many operons contain genes that are not homologous, but have some kind of functional link. As a general mechanism of operon formation, the Natal Model is inadequate.

The Fisher Model postulates that clustering of genes into operons offers the benefit that random recombination events will tend to separate co-adapted genes less often if they are clustered together. This model has suffered criticism recently because of observations of orthologous replacement *in situ *of operon genes [13,14] which suggests that the primary reason for operon formation is unlikely to be the preservation of co-adapted alleles.

The Co-regulation Model [15] states that operons are formed in order to facilitate the production of gene products in equal measures. This theory only accounts for operon maintenance. In order for an operon to spontaneously form, rare, highly specific recombination events must occur. However, it has recently been asserted that operon formation is driven by co-regulation [16]. This assertion is largely due to the more complex regulatory regions associated with operons in some gamma-proteobacteria compared with genes that are not in operons. However, this study only focused on operons and not on the broader issue of cluster formation.

The Selfish Operon Model (SOM) suggests that operons in prokaryotes are in some respect like viruses or transposons and their formation facilitates their horizontal gene transfer (HGT) [12]. The formation of an operon is therefore of no direct benefit to the organism but it means that the fitness of gene cluster itself is enhanced. An extension of the SOM posits that if HGT is indeed the main reason for operon formation, non-essential genes are more likely to be in operons/clusters than essential genes [12]. However, Pál and Hurst [17] have provided evidence that essential genes are more likely to be found in operons and clusters than non-essential genes, thereby presenting a significant problem to the SOM.

Lastly, a recent proposition has been made that gene clustering is due to the relative difficulty of protein movement through the cellular matrix [18]. This model, known as the Protein Immobility Model (PIM), suggests that because transcription and translation are coupled in prokaryotes, the resulting physical proximity of enzymes minimizes the steady state level of reaction step intermediates thereby saving energy and reducing the amount of protein that needs to be produced. The PIM has not been tested using empirical data, but has been supported by computer simulation. An observation that indirectly supports the PIM is the study by Elowitz *et al *[19] that shows that protein diffusion is slower through the cytoplasm than through water, is adversely affected by the size of the protein, and is also reduced when expression levels are higher.

Because paa genes show a patchy phylogenetic distribution and previously observed paa clusters have diverse structures that appear to be independent of the species phylogeny, we felt that this pathway was important to study from an evolutionary standpoint. Indeed, phenylacetate degradation has previously been identified as a potential model for understanding the evolution of metabolic pathways [20]. By examining the gene content of previously studied paa clusters a total of 17 genes are associated with the pathway including catabolic genes, regulatory genes, a transporter and an exporter. In this study we identify new paa gene clusters and examine the structure and distribution of paa gene clusters with respect to their evolution and implications for models of both cluster and operon formation.

## Methods

### Homolog identification

We implemented an iterative strategy for locating homologs to all 17 genes encoding proteins involved in the degradation of phenylacetate. Initially, the genomes for taxa containing known *paa *gene clusters, previously reported in the literature, were downloaded from GenBank [21]. The list of genomes used in the initial search with known *paa *gene clusters can be found in the supplementary information . We used a BLAST-based [22] similarity search strategy where we extracted all the known *paa *genes from these initial genomes and used them in order to find homologs in other completed bacterial genomes. These additional bacterial genomes were downloaded from GenBank, bringing the total number of genomes in the dataset to 102.

We generated alignments using ClustalW 1.81 [23] for genes where we found multiple homologs. The exceptions were the *paaL *and *paaM*, genes that were only found only in *P. putida KT2440*. This gave a total of 15 initial alignments. These alignments were then used as input for PSI-BLAST using the default parameters [22], with the larger dataset of 102 bacterial genomes as the input database. This gave us a comprehensive list of homologs.

We wrote a number of PERL scripts (available on request) to cross-reference the result files generated from the PSI-BLAST searches and identify clusters of genes from this pathway that were co-located on their respective genomes. If two genes found in the result files generated by the PSI-BLAST searches came from the same genome and had no more than five intervening genes between them, then such genes were considered to be an initial linked pair. All initial linked pairs were identified and then manually merged if they overlapped. In this way, clusters of various sizes were identified.

### Construction of phylogenetic trees

Each of the 15 gene families were used to build phylogenetic trees. The amino acid sequences of all homologs were extracted from their genome files and each family was aligned using Muscle v3.5 [24] with all settings at their default values. Model selection was performed on the alignments using ModelGenerator [25] and maximum likelihood phylogenetic trees were constructed based on the selected models using Phyml v3.0 [26]. Confidence in phylogenetic hypotheses was assessed using the bootstrap resampling approach [27].

### Visualisation of clusters on phylogenetic trees and operon identification

For each gene family, we wished to visualise both the relationships among members of the family and their cluster context simultaneously. Visualisation of each gene cluster was achieved by extracting the necessary genomic location information for the cluster from the corresponding GenBank file. This was carried out automatically using PERL scripts. Once this information was parsed from the GenBank file, the corresponding cluster was drawn using the postscript language (Adobe Systems, San José, California). If, for instance, a cluster contained the genes *paaA *and *paaB*, then this cluster will appear on the *paaA *tree at the phylogenetic position of the *paaA *gene and on the *paaB *tree at the phylogenetic position of the *paaB *gene. Operons were identified using the MicrobesOnline database . Know operons were crosschecked with the predictions to access quality of the predictions in the database.

## Results

In order to test whether a cluster has been independently assembled more than once, we can examine the phylogenetic trees of both cluster and non-cluster homologs. If a cluster has originated once and has never been subsequently perturbed, then for every gene in the cluster the corresponding phylogenetic tree will include a clade containing all the species in which the cluster is present. Given the prevalence of HGT [28] this clade does not have to correspond to any recognized phylogenetic group. The only relationships that are of importance are the relationships of the genes.

### Variation in cluster and operon content and context

**Figure 2:**
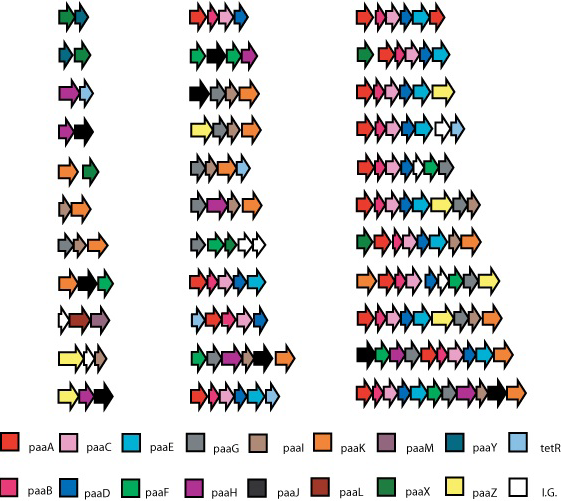
**An exhaustive list of all observed operons in the dataset of 102 genomes examined**. Each arrow represents a gene, with the name of the gene being given in the legend. I.G. refers to an intervening gene, which is a gene in the cluster that is not involved in the degradation of phenylacetate.

Table 1 shows a summary of all 1,311 homologs identified via the PSI-BLAST searches, in terms of the frequency with which they were found in a *paa *gene cluster and if found in a cluster, how often they were in an operon. Figure 2 contains a complete list of all observed unique operons in our dataset. These data together detail the considerable variation across genes in terms of their tendency to be found in a cluster or operon.

**Table 1:** Frequency of presence in a cluster and operon.

**Gene**	**In cluster**	**Not in cluster**	**In operon**	**Not in operon**	**Total genes**
paaA	25	0	25	0	25
paaB	25	0	25	0	25
paaC	25	0	25	0	25
paaD	25	0	25	0	25
paaE	19	4	19	0	23
paaF	14	163	10	4	177
paaG	17	6	16	1	23
paaH	12	133	9	3	145
paaI	19	0	16	3	19
paaJ	11	266	11	0	277
paaK	21	16	19	2	37
paaX	11	2	7	4	13
paaY	5	79	3	2	84
paaZ	22	377	8	14	399
tetR	12	2	7	5	14
**All**	**263**	**1048**	**225**	**38**	**1311**

In the cases of *paaA*, *B*, *C *and *D *the genes were always found in an operon and obviously therefore, always in a cluster. For *paaE*, in 19 out of 23 instances it was found with other *paa *genes. *paaI *was always found in a cluster (19 occasions) and the majority of times (16 out of 19), in an operon. Similarly *paaX *and *tetR *were found relatively rarely (13 and 14 times respectively) and were usually found in clusters (11 out of 13 for *paaX*, 12 out of 14 for *tetR*) and 7 times each, they were in operons. *paaG *was found 23 times, 17 times in a cluster and 16 out of those 17 times it was found in an operon. *paaK *is found 37 times and in slightly more than 50% of the instances (21 of 37), it is in a cluster and the majority of times that it is in a cluster it is in an operon (19 of 21). The remaining five genes *paaF, paaH, paaJ, paaY and paaZ *are more widely distributed and the majority of times these homologs are not found in clusters or operons. The gene that is least likely to be found in an operon is *paaY*, which is only found in an operon in 3 out of 84 instances. Interestingly, apart from *paaA, B, C, D *and *E *where being in a cluster automatically means being in an operon, most other genes are found in an operon the majority of the times they are found in a cluster. The exception is *paaZ*, where for 14 out of 22 instances of the gene being in a cluster it is not in an operon.

Figure 2 shows the set of unique operons involving two or more *paa* genes found in all identified clusters. The most striking aspect of this analysis is the sheer diversity in terms of size, gene content and gene order among the operons. A total of 33 different operons were identified, ranging in size from 2 to 11 genes. Out of the 33 unique operons only two display identical gene content, one being *paaXY*, the other *paaYX*. This diversity is not surprising from a mathematical standpoint, given that 17 genes were examined in the study. Even operons consisting of only 2 genes there are 289 possible permutations. Aside from the *paaABCDE* operon, which is clearly under strong selection (all 25 clusters form operons), no particular operon composition or configuration is dominant. This result seems to indicate that operon formation (apart from *paaABCDE*) is not dependent on the composition of the genes that are present. Operons seem to form, simply when members of the pathway are present and no single operon composition or order is obligatory.

### Analysis of gene clusters containing all 11 catabolic paa genes

In order to establish how operons and clusters grow we chose to focus on the largest clusters. We wished to analyse whether for large clusters there was selection to keep co-adapted alleles together. We identified five clusters in the dataset that were almost complete and were present in genomes that were not thought to be each others' closest relatives as judged using phylogenetic supertree methods based on completed genomes [29]. These included the clusters found in *E. coli*, *P. putida, Rhodoccoccus sp., Nocardia farcinica *and *Corynebacterium efficiens*. The evolutionary history of these clusters was examined in detail: phylogenetic trees and additional data are available as supplementary information .

Figure 1 shows the operon structures observed in *E. coli *and *P. putida*. In *E. coli K12*, all fourteen genes involved in the pathway are clustered together and the cluster is broken into three operons [3]. *paaABCDEFGHIJK *are present in one operon, *paaXY *in another and the paaZ gene is transcribed by itself.

Superficially, the cluster in *P. putida *has high levels of similarity to the cluster in *E. coli K12 *with simple rearrangements of the order of blocks of genes accounting for the majority of the observed differences, at first glance (see figure 1). In *P. putida *the gene cluster is arranged in five operons [3] with *paaABCDE *being in one operon and *paaFGHIJK *being in a second, where both are merged in *E. coli*. *paaLM *and an unrelated gene are in another operon, *paaYX *is in an operon (the order is reversed in *E. coli*) and *paaZ *is transcribed by itself in the cluster. The gene content difference between the two clusters is the presence of *paaL*, a phenylacetic acid transporter, and *paaM*, a phenylacetic acid specific porin, along with an additional gene not known to be involved in phenylacetate degradation. *paaL *and *paaM *are only present in *P. putida *and in none of the other 102 genomes studied.

The phylogenetic analyses of the genes in these two clusters reveals a much greater degree of difference. We examined the phylogenetic trees for all genes in these clusters, expecting that the individual genes would be each other's closest relatives or at least reasonably closely related. We found that indeed for the *paaA, C, D, F, G, I, J, K and X *genes the *E. coli *and the *P. putida *copies grouped closely on a phylogenetic tree. Contrastingly, for *paaB, E, H, Y *and *Z *we see support for the separation of the two *E. coli *sequences from the *P. putida *sequences on their respective phylogenetic trees. This result indicates that orthologous gene displacement has replaced a considerable number of genes in these clusters since the clusters separated from their common ancestor. Given the compositional similarity the most parsimonious explanation is that a complete cluster existed in the past and the two that we see in *E. coli *and *P. putida *today are descended with great modification, probably by rearrangement, insertion and orthologous displacement from the ancestral cluster. Of particular interest is the *paaABCDE *operon which is relatively invariable (see previous results), but from this analysis it is still subject to gene turnover and replacement. We note that these are the two most complete and similar clusters in our dataset. If we extrapolate from this result and go further back through evolutionary history assuming a similar rate of gene replacement, then it is likely that replacement of every single gene in this cluster – one at a time – can occur relatively rapidly.

### The *Rhodococcus sp./Nocardia farcinica/Corynebacterium efficiens *clusters

*Rhodococcus sp*. and *Nocardia farcinica *have two clusters that are very similar both in terms of gene content and orientation of genes within the cluster. In all phylogenetic analyses of the *paa *genes in the clusters, we find strong support for a sister group relationship between these two taxa. This suggests a recent common ancestor of both clusters. The *N. farcinica *cluster is split into four operons, the first is *paaI *by itself, the second contains a non-*paa *gene and *paaZ*, the third is *tetR *by itself and the fourth contains paa *J, F, H, G, A, B, C, D, E *and *K*. The *Rh. sp*. cluster is split into two operons, the difference being that *paaI *is in an operon with a non-paa gene and paaZ. This is followed by an operon consisting of *paaJ, F, H, G, A, B, C, D, E*, and *K*. These clusters are very different in terms of gene order when compared with either *E. coli *or *P. putida*.

The *Corynebacterium efficiens *cluster has some similarities to the *Rh. sp./N. farcinica *cluster. Firstly the gene content is almost identical, the only difference being that there are two copies of *paaF *in the *C. efficiens *cluster while *paaG *is absent. Secondly, all three clusters contain a gene of unknown function, and these three genes are homologs of one another. Thirdly, the *C. efficiens *cluster contains a copy of the *tetR *transcriptional regulator, as does the *N. farcinica *cluster. Lastly, there are subtle patterns of similarity in gene order with *paaZ, J, G, F *and *H *all in close proximity to one another in the three clusters, as were *paaA, B, C, D, E *and *K*.

When we reconstructed the phylogenetic relationships between the genes on the *C. efficiens *and the *Rh. sp./N. farcinica *clusters we found that a sister group relationship was recovered for the *paaF, H, I, J, and K *genes with strong bootstrap support for this arrangement. However, for the *paaA, B, C, D, E and Z *genes there is strong support for grouping *Rh. sp./N. farcinica *with *Streptomyces coelicolor*, although in some cases the *C. efficiens *homolog is nearby on the tree. *S. coelicolor *has a *paa *cluster consisting of *paaK, I, A, B, C, D *and *E*. The results suggest that the *paaABCDE *operon in *Rh. sp./N. farcinica/S. coelicolor *are each others closest relatives for all the genes in the operon, while for the *paaK *and *paaI C. efficiens *groups while *Rh. sp*. and *N. farcinica*, to the exclusion of *S. coelicolor*.

An analysis of all five near-complete clusters does not support a single origin of these clusters and there are no genes that place *E. coli *or *P. putida *as sister-taxa to genes from the *C. efficiens *or *Rh. sp./N. farcinica *genes. This indicates that formation of these near-complete clusters occurred independently on at least these two occasions, one assembly occurring in the proteobacteria and the other in the actinobacteria.

**Figure 3:**
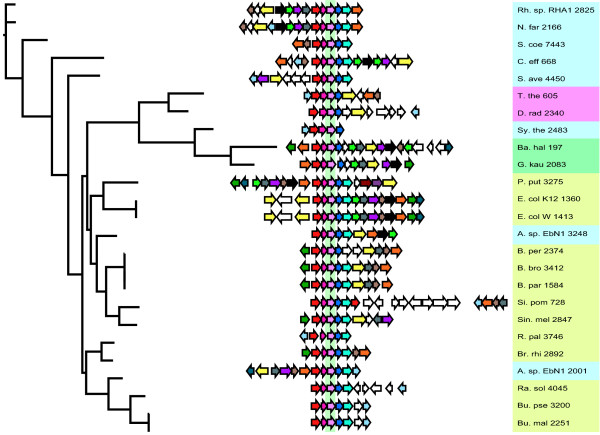
**On the left is the gene tree for *paaC*, in the middle are the clusters of genes in which the respective *paaC *genes are found, with the *paaC *genes aligned to one another and facing away from the tree**. On the right are the organism abbreviations (see supplementary information for list of organisms and abbreviations) and the gene number for the *paaC *gene, which indicates how many genes between it and the origin of replication for the genome in which it is found.

**Figure 4:**
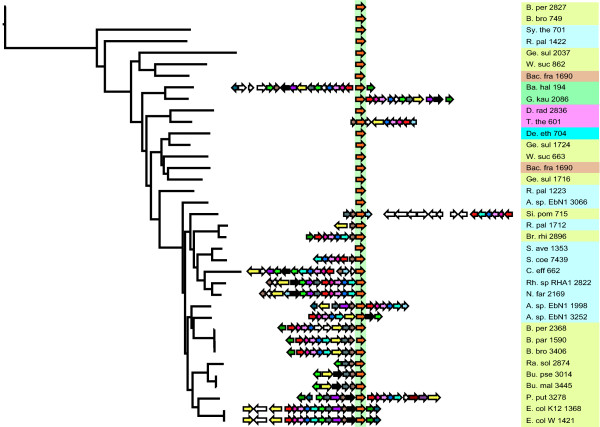
**The same layout as figure 3, but the information represented is based upon the paaK gene**.

A comparative analysis of the evolutionary histories of the *paaK *and the *paaC *genes can be seen in figures 3 and 4. The *paaC *gene is always found in an operon with *paaA*, *B *and *D*. Also, there is only one instance where this operon is not found in a cluster with other genes from the phenylacetate degradation pathway (i.e. in the case of *Symbiobacterium thermophilum*). The *paaK *gene is found in a cluster of more than two phenylactetate degradation genes approximately half of the times it is observed, the rest of the time, it is found as a single gene in the genome. There are four clans [30](the tree is only rooted for convenience, but is really unrooted) in which the *paaK *gene is at the edge of a cluster. Overall, it can be seen that the clusters for both genes dynamically grow, shrink and are rearranged (additional phylogenetic trees for every gene are supplied in the supplementary information and the reader should consult these trees).

To illustrate the variability in cluster context it is possible to take some examples from figure 3. The numbers that follow the taxon names are indicative of the physical location of the gene in the genome. In the *paaC *tree (figure 3), the two instances of this gene in *Azoarcus sp. EbN1 *are genes number 2001 and 3248. These genes are spaced approximately 1250 genes apart on the *A. sp. EbN1 *genome, both genes are in a paa cluster and they are not particularly closely-related genes as evidenced by their phylogenetic positions. A reasonable speculation is that one or both of these genes was introduced into the genome via horizontal gene transfer. In contrast the two instances of *paaK *(figure 4) found in *A. sp. EbN *(labelled *A. sp. EbN1 *3252 and *A. sp. EbN1 *1998 on the *paaK *tree) are indeed each others closest relatives, indicating a relatively recent gene duplication event. The *Thermus thermophilus *and *Deinococcus radiodurans *genes on both trees are nearest neighbors, suggesting a relatively recent common ancestor. This relative recentness of common ancestry might lead us to expect that the cluster context of these two genes might be similar, however, the *D. radiodurans paaK *gene is not in a cluster, whereas the *D. radiodurans paaC *gene is in a cluster.

On the *paaC *tree, there are three *Bordetella *clusters that are almost identical in terms of gene content and order. However, in one of the three genomes (that of *B. pertussis *Tohama I) there are two genes in the middle of the cluster that are not found in the other two strains. These two genes seem to have displaced the *paaE *gene in *B. pertussis *Tohama I, which lacks a copy of *paaE*. The other two *Bordetella *strains have copies of *paaE *in their clusters. The most parsimonious reconstruction, based on the *paaC *tree is that these two genes have been inserted into the cluster in *B. pertussis *Tohama I (see supplementary information for more gene trees).

These observations demonstrate the enormous variability and rapid rate of assembly and disassembly of clusters as well as the semi-independent assembly of two near-complete clusters.

## Discussion

In this work, we have analysed the evolutionary history of the genes involved in the phenylacetate degradation pathway, with a view to understanding the origin and spread of functionally-related gene clusters and operons.

The most surprising result from this study is the diversity we see in terms of both cluster and operon structure. We observe that the clustering of phenylacetate degradation genes has occurred repeatedly in several different lineages, the clusters themselves are mosaics and are generally composed of genes that have been acquired from other species, either recently or relatively recently. Often, strains of the same species have very different cluster structures and indeed in the case of *E. coli *and *P. putida*, even though the clusters look similar, many of the genes cannot trace their most recent common ancestor to the same point. In other words, orthologous gene displacement is quite common, as is illegitimate recombination. This has been reported previously [13] and it indicates that the selective pressure to form clusters is not so strong that clusters, once formed, become immutable or that clusters continue to become larger.

In general, operon destruction as well as operon formation are seen to occur in our dataset and we observe a total of 33 unique operon structures. This suggests that either the selective advantage that accrues as a result of operon formation is not very strong and recombination followed by random genetic drift can successfully break up operons (a neutralist explanation) or that if indeed a selection pressure exists that drives operon formation, there exists another opposing selection pressure to split operons. It is also possible that a selective advantage could exist to create an operon, but subsequently this advantage is no longer present as the environment changes. Irrespective of the explanation, it seems that for this particular pathway, the formation of large operons containing most or all of the genes is not necessarily hugely important, or perhaps it is not possible. The exception to the rule is seen in the *paaABCD *operon, which is strongly conserved. The obvious explanation is that these four protein products physically interact and their existence in equimolar concentrations is necessary. Therefore, there is a gradient of selective pressure for co-regulation which is strongest for interacting proteins in our small dataset, less strong for proteins that do not physically interact and indeed co-regulation might be a selective disadvantage in some cases (in 14 out of 22 cases *paaZ *is in a cluster but not in an operon) and may lead to the successful destruction of an operon.

The study also sheds some light on the various models of cluster and operon formation. We expect from the Natal Model of operon growth that all genes in the operon are evolutionarily related. This theory is clearly insufficient to account for the observations in this analysis.

The Selfish Operon Model (SOM) posits that operons exist so that they can be easily transferred via horizontal gene transfer. Our analysis shows that there is a evidence of gene replacement within a cluster and within an operon and this presents a difficulty with the hypothesis that operons exist in order to facilitate their transfer as a group. Additionally, the sheer diversity of operons present in the analysis is at odds with the SOM. We see 33 unique operon structures. Even the clusters of *E. coli K12/W *and *P. putida*, which are clearly homologous, differ in gene content, order, operon structure and show evidence of orthologous replacement via HGT. While it is not in doubt that there is an advantage to passing a set of genes horizontally, our results show little evidence of selfish operon style transfers. The only stable operon structure is that of *paaADCDE *and this is an example of an operon that cannot exist outside of a selfish operon framework, since the gene products form a complex with one another. In addition, Pál and Hurst [17] have already shown that essential genes are more likely to be in an operon than non-essential genes and this is also incompatible with the SOM.

The Fisher Model states that cluster formation is a way of keeping co-adapted alleles together. It is clear from our analysis that the turnover rate of alleles is high and alleles do not seem to spend much time being inherited together and so this model is not compatible with our observations.

The Co-regulation Model, while recently receiving some support from an analysis of operons only [16] is also insufficient to cover our observations. We see genes present in a cluster, but not in an operon 38 times. We see genes in operons 225 times, however, 119 of those times the operon is the *paaABCDE *operon, which contains genes that form a single heteromeric complex. The Co-regulation Model only governs operon maintenance and is strongly in operation for the maintenance of *paaABCDE *but is still insufficient to explain all our data.

The Protein Immobility Model (PIM) fits with the idea that there is a small selective advantage for clustering genes together. The reason for this small selective advantage is the effect macromolecular crowding has on the movement of proteins in the cell. Macromolecular crowding tends to increase the speed of biochemical reactions [31], whilst simultaneously limiting the ability of large proteins to move around the cell. While the cellular matrix is a dynamic environment, the movement of a protein through the cytoplasm of a prokaryote is slower than through water [19] and when several proteins are involved, this is likely to result in sufficient restriction of movement that a selective advantage accrues for an organism that synthesizes functionally related proteins in close proximity to one another. However, the PIM only covers the formation of clusters and does not cover operon formation and maintenance. We would see operon formation and maintenance to be a not-inevitable consequence of cluster formation, perhaps simplifying transcription.

A number of studies have indicated that transcriptional control of independent transcription units (single genes and operons) is likely to have influenced genomic structure [32]. This is reflected in the co-localisation of genes that are controlled by the same transcription factor. Additionally, the distribution and orientation of transcription units is not random [33] and is associated with an optimization process. In this study, we have shown that while these genome optimization processes are under way, the process of horizontal gene transfer and within-cluster gene content perturbation is continuous and at times fairly radical.

We note that no one model of operon assembly completely covers our observations. Perhaps a more robust model would be one that deals with cluster and operon formation as different levels of organisation. We know that operon formation occurs subsequent or at the same time as cluster formation, however, our data clearly show that operon formation is not absolutely necessary. The majority of genes in clusters do become involved in operons, but this is likely to be a secondary advantage ensuring that they are transcribed at the same time. We feel that a comprehensive model requires a component that provides a selective advantage for moving genes closer together in a genome and a separate component providing selective advantage for operon formation. In terms of the current models, the best fit would be a combination of the PIM and the Co-regulation Model.

Perhaps more important, however, is evolutionary history of the genes of the phenylacetate degradation pathway. The massive diversity of the clusters and operons observed, coupled with complete lack of correlation to phylogeny, provides an interesting insight into just how dynamic is the process rearranging the position of genes in a genome. While this is only a single pathway, the evidence still strongly implies the existence of a complicated underlying system in prokaryotes based upon a recombination selection balance. Even if phenylacetate degradation is unusual when compared to clusters associated with amino acid biosynthesis or other core pathways, it may provide a much deeper understanding of the principles of cluster and operon formation than static, widely distributed gene clusters ever could.

## Conclusion

We conclude that the Selfish Operon Model is insufficient to account for the assembly of clusters and operons and that any relevant model for cluster and operon assembly needs to include a credible component that provides a selective advantage for genes moving closer together. The best fitting model would combine the PIM and the Co-regulation Models.

## Authors' contributions

FJM and JMcI designed the project. FJM wrote software and analysed the data. FJM and JMcI interpreted the results and wrote the manuscript.
